# Global Analysis of Neuronal Phosphoproteome Regulation by Chondroitin Sulfate Proteoglycans

**DOI:** 10.1371/journal.pone.0059285

**Published:** 2013-03-18

**Authors:** Panpan Yu, Trairak Pisitkun, Guanghui Wang, Rong Wang, Yasuhiro Katagiri, Marjan Gucek, Mark A. Knepper, Herbert M. Geller

**Affiliations:** 1 Developmental Neurobiology Section, Cell Biology and Physiology Center, Division of Intramural Research, National Heart, Lung, and Blood Institute, National Institutes of Health, Bethesda, Maryland, United States of America; 2 Epithelial Systems Biology Laboratory, National Heart, Lung, and Blood Institute, Division of Intramural Research, National Institutes of Health, Bethesda, Maryland, United States of America; 3 Proteomics Core Facility, Division of Intramural Research, National Heart, Lung, and Blood Institute, National Institutes of Health, Bethesda, Maryland, United States of America; 4 Center for Biologics Evaluation and Research, U.S. Food and Drug Administration, Bethesda, Maryland, United States of America; Hertie Institute for Clinical Brain Research, University of Tuebingen., Germany

## Abstract

Chondroitin sulfate proteoglycans (CSPGs) are major components of the extracellular matrix which mediate inhibition of axonal regeneration after injury to the central nervous system (CNS). Several neuronal receptors for CSPGs have recently been identified; however, the signaling pathways by which CSPGs restrict axonal growth are still largely unknown. In this study, we applied quantitative phosphoproteomics to investigate the global changes in protein phosphorylation induced by CSPGs in primary neurons. In combination with isobaric Tags for Relative and Absolute Quantitation (iTRAQ) labeling, strong cation exchange chromatography (SCX) fractionation, immobilized metal affinity chromatography (IMAC) and LC-MS/MS, we identified and quantified 2214 unique phosphopeptides corresponding to 1118 phosphoproteins, with 118 changing significantly in abundance with CSPG treatment. The proteins that were regulated by CSPGs included key components of synaptic vesicle trafficking, axon guidance mediated by semaphorins, integrin signaling, cadherin signaling and EGF receptor signaling pathways. A significant number of the regulated proteins are cytoskeletal and related proteins that have been implicated in regulating neurite growth. Another highly represented protein category regulated by CSPGs is nucleic acid binding proteins involved in RNA post-transcriptional regulation. Together, by screening the overall phosphoproteome changes induced by CSPGs, this data expand our understanding of CSPG signaling, which provides new insights into development of strategies for overcoming CSPG inhibition and promoting axonal regeneration after CNS injury.

## Introduction

Chondroitin sulfate proteoglycans (CSPGs) are a family of extracellular matrix (ECM) molecules that contribute to the failure of axon regeneration following injury to the adult mammalian central nervous system (CNS) [1]. In primary neuronal cell culture, CSPGs strongly inhibit neurite outgrowth of different types of neurons such as dorsal root ganglion (DRG) neurons [2], cerebellar granule neurons (CGNs) [3] and retinal ganglion cells (RGCs) [4]. In experimental animal models of spinal cord injury, enzymatic degradation of CSPGs with chondroitinase ABC promotes axonal regeneration and improves behavioral outcomes [5], [6], [7]. CSPGs have been one of the important targets for promoting axonal regeneration in the injured CNS.

CSPGs are comprised of a protein core with one or more covalently attached chondroitin sulfate glycosaminoglycan (CS-GAG) side chains. Much evidence shows that the inhibitory actions of CSPGs depend on the specific sulfation patterns in CS-GAG chains [5], [8], [9], whereas several reports suggest that CSPG core proteins can also exert inhibitory effects on neurite outgrowth independent of CS-GAG chains [4], [10]. The molecular mechanisms by which CSPGs restrict axonal growth are not well understood. For many years, CSPGs had been thought to exert their inhibition through blocking the interactions of growth cones with growth promoting extracellular matrix (ECM) and cell adhesion molecules. Most recently, four receptors for CSPGs have been identified: two members of the receptor protein tyrosine phosphotase (RPTP) family, RPTPσ [11], [12] as well as the family member LAR [13], and the Nogo receptor (NgR) family members NgR1 and NgR3 [14]. So far, several signaling pathways have been reported to mediate CSPG inhibition on neurite growth, including the protein kinase C [15], Rho/ROCK signaling [16], [17] and Akt-GSK3β pathways [18]. Given the diversity and complexity of CSPGs both in structure and in their binding properties, intracellular signaling cascades are expected to be complex.

Reversible protein phosphorylation is one of the most important posttranslational modifications for cellular regulation and signal transduction in eukaryotic cells. Protein mass spectrometry has emerged as a key technology for screening protein posttranslational modifications including phosphorylation. It allows simultaneous phosphorylation site mapping and quantitation in a single experiment. The iTRAQ technology enables the comparison of up to eight different samples in one mass spectrometry-based experiment [19], [20]. The aim of this study was to profile global phosphorylation changes in primary neurons induced by CSPGs. By applying an iTRAQ-based quantitative phosphoproteomics strategy, we identified a group of differentially phosphorylated proteins which implicate a number of signaling pathways that are regulated downstream of CSPGs. These pathways and proteins may serve as targets for preventing the actions of CSPGs on axonal regeneration.

## Results

### Phosphoproteomic Profiling of Primary Neurons in Response to CSPGs

**Figure 1:**
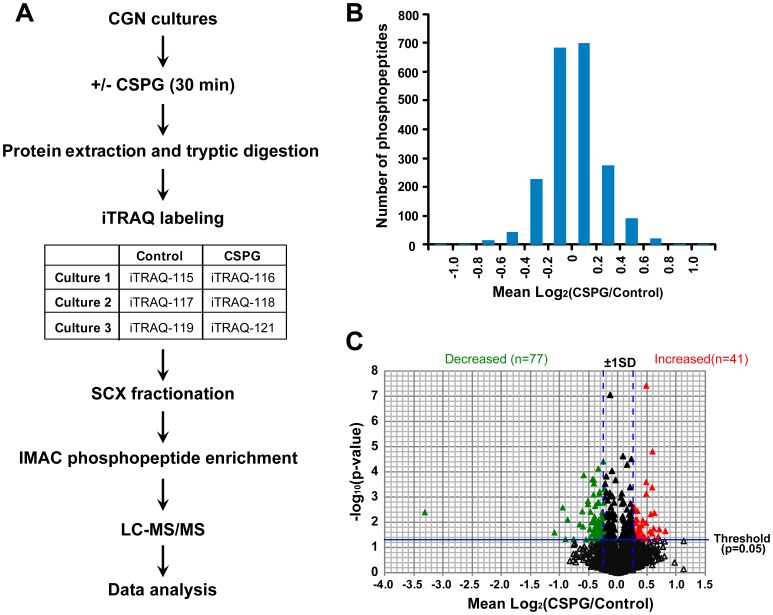
Overview of phosphoproteomic profiling. (**A**) Strategy used for iTRAQ-based quantitative phosphoproteomics of CSPG-treated mouse CGNs. *CGN*, cerebellar granule neuron; *SCX*, strong cation exchange chromatography; *IMAC*, immobilized metal affinity chromatography. (**B**) Distribution of changes for all quantified phosphopeptides. (**C**) A volcano plot shows the differentially regulated phosphopeptides. The horizontal line marks the threshold at1.301 = -log (*p = *0.05) and the vertical dash lines represent the positions with ratios of Mean+1SD and Mean-1SD. Using the dual criterions of p<0.05 and change>1SD, 77 phosphopeptides were defined as decreased (green) and 41 were increased (red) by CSPGs.

To monitor CSPG-induced regulation of protein phosphorylation, three pairs of cell lysates with or without CSPGs treatment were collected from three independent primary CGN cultures and subjected to iTRAQ-based quantitative phosphoproteomic analysis as described in methods. A work flow of sample processing and analysis is presented in Fig. 1. To expand the size of the identified phosphoproteome, samples were fractionated using SCX chromatography before phosphopeptide enrichment via IMAC. The peptide samples representing 26 SCX fractions were analyzed on a Thermo LTQ Orbitrap Velos mass spectrometer. The resulting MS spectra were matched to specific peptide sequences using both the Mascot and the SEQUEST algorithms. The dataset was filtered for a false discovery rate <1% by target-decoy analysis. A total of 2214 unique phosphopeptides corresponding to 1118 phosphoproteins were identified. 70% of peptides identified were phosphopeptides, with IMAC enrichment efficiency ranging from 40% to 100% for individual fractions. As is typically seen, the majority of the phosphopeptides were phosphorylated on serines or threonines with only 1.4% phosphorylated on tyrosines.

Changes in the phosphorylation level of the phosphopeptides were quantified based on the intensity of different iTRAQ reporter ions. Among 2214 phosphopeptides identified, 1988 phosphopeptides were quantified in all three biological replicates; 2064 phosphopeptides were quantified in at least two of the three biological replicates. The complete list of all quantified phosphopeptides is shown in Table S1. Of those phosphopeptides quantified, most were statistically unchanged in abundance in response to CSPG treatment. The distribution of all quantified ratios is shown in Fig. 1B and C. The log_2_(CSPG/Control) ranged from −3.32 to 1.13, with an average log_2_(CSPG/Control) = 0.018±0.24 (Mean±SD). There were 511 phosphopeptides changed with mean log_2_(CSPG/Control) values either greater than 0.262 (Mean+1SD) or less than −0.225 (Mean-1SD). Of these 511 phosphopeptides, 118 had p<0.05 in abundance upon exposure to CSPG. A complete list of these 118 phosphopeptides which meet the dual criteria of p<0.05 and 1SD cutoff are shown in Table S2, with 41 increased and 77 decreased in phosphorylation by CSPGs. A subset of 29 representative phosphopeptides from this list are shown in Table 1.

**Table 1:** List of phosphopeptides that show significant changes in abundance in response to CSPG treatment with mean log_2_ (CSPG/Control) >0.5 or<−0.5.

Peptide sequence	Accession	Protein Name	Mean	*p*-value	Phosphosite
ENAS*PAPGTTAEEAMSR	NP_476512	Large proline-rich protein BAG6	0.82	0.022	S995
LQLDGSLTLNSSSSSLQAS*PR	NP_853625	Enhancer of mRNA-decapping protein 4	0.71	0.019	S680
SS*PVCSTAPVETEPK	NP_083930	Autophagy-related protein 2 homolog B	0.70	0.036	S240
TQVLSPDS*LFTAK	NP_034936	Afadin	0.63	0.048	S1722
QAS*PLGTPTPEADTTLLK	NP_542764	GTP-binding protein REM 2	0.63	0.004	S27
FPPPQELSQDS*FGSQASSAPSMTSSK	NP_001074288	AT-rich interactive domain-containing protein 1A	0.62	0.017	S608
MEANGS*PGTSGSANDSQHDPGK	NP_473384	RNA-binding protein Musashi homolog 2	0.59	0.000	S6
VEEEPIS*PGSTLPEVK	NP_203538	Dedicator of cytokinesis protein 2	0.59	0.027	S1683
SVS*PGVTQAVVEEHCAS*PEEK	NP_032660	Microtubule-associated protein 1B	0.59	0.000	S1293, S1307
S*AESLQSLNSGLCPEK	NP_001028389	Prickle-like protein 1	0.57	0.005	S592
AMGS*GGAGSEQEDTVLFRR	NP_035550	Survival motor neuron protein	0.55	0.003	S5
SLVSPIPSPTGTISVPNSCPAS*PR	NP_951031	Forkhead box protein K1	0.54	0.024	S243
VSEEAESQQWDTSKGDQVSQNGLPAEQGS*PR	NP_787030	Spectrin beta chain, brain 1	0.54	0.014	S2137
NAEEES*ESEAEEGD	NP_080100	Basic leucine zipper and W2 domain-containingprotein 1	0.52	0.043	S411
NEEDEGHSNSS*PR	NP_031542	Heterogeneous nuclear ribonucleoprotein D0	−3.32	0.004	S83
GDS*DDEYDRR	NP_113582	Serrate RNA effector molecule homolog	−1.09	0.025	S4
IESPLET*LSAQNHSASMTEVT	NP_848871	Lipid phosphate phosphatase-related protein type 1	−0.95	0.003	T311
AS*PVADASRR	NP_892041	Zinc finger protein 692	−0.90	0.009	S3
NRHS*PDHPGMGSSQASSSSSLR	NP_080161	Mediator of RNA polymerase II transcriptionsubunit 19	−0.86	0.008	S226
RAS*DGGANIQLHAQQLLK	NP_081774	Serine/threonine-protein kinase SIK3	−0.76	0.046	S551
VAPGPSSGCTPGQVPGSS*ALSSPR	NP_775613	Neuron navigator 1	−0.75	0.016	S1376
GHAGGQRPEPSS*PDGPAPPTR	NP_542764	GTP-binding protein REM 2	−0.65	0.012	S296
SFS*MQDLTTIRGDGAPAPSGPPPPGTGR	NP_848723	Receptor expression-enhancing protein 1	−0.61	0.034	S152
SSS*YSEANEPDLQMANGSK	NP_001001602	Disabled homolog 2-interacting protein	−0.61	0.004	S663
SRGS*SAGFDR	NP_036098	Proteasome subunit alpha type-6	−0.60	0.014	S5
AMS*TTSVTSSQPGK	NP_038838	Drebrin-like protein	−0.59	0.000	S274
ELS*QVLTQR	NP_001032850	F-actin-capping protein subunit beta	−0.54	0.048	S263
AGGAS*PAASSTTQPPAQHR	NP_848872	Interferon regulatory factor 2-binding protein 1	−0.51	0.001	S453
VTSSVPLPSGGTS*SPSR	NP_710147	Sorting nexin-17	−0.51	0.002	S335

### Functional Analysis

**Figure 2:**
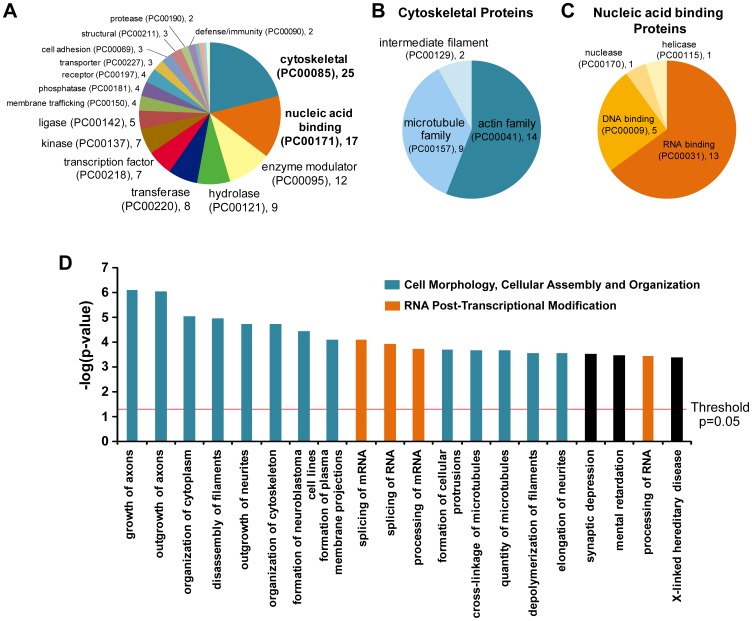
Protein classification of significantly changed phosphoproteins. (**A**) Gene ontology analysis of changed phosphoproteins using PANTHER program. (**B**) Subdivision of the category cytoskeletal protein (PC00085) in (A). (**C**) Subdivision of the category nucleic acid binding (PC00171) in (A). (**D**) Significance of biological functions refers to the –log (p-value) obtained by the Ingenuity program. The top 20 biological functions with significant enrichment are shown. Threshold is at 1.031 = -log (p = 0.05).

The set of 118 significantly changed phosphopeptides were annotated in the gene ontology format using the PANTHER classification system. The top three categories were cytoskeletal proteins (n = 25), nucleic acid binding proteins (n = 17) and enzyme modulators (n = 12) (Fig. 2A). Among the cytoskeletal proteins, 14 were actin family cytoskeleton proteins including motor and non-motor actin binding proteins; 9 were microtubule family cytoskeleton proteins including motor and non-motor microtubule binding proteins (Fig. 2B). Among the nucleic acid binding category, 76.5% were RNA binding proteins including mRNA processing factors (n = 5), ribonucleoproteins (n = 2), ribosomal proteins (n = 2) and translation factors (n = 1) (Fig. 2C).

To further interpret the data in a biological context, the list of proteins whose phosphorylation was significantly changed was analyzed using the Ingenuity program which identifies networks and biological functions which are most significant to the data set. The top two networks involved were (i) Cell Morphology, Cellular Assembly and Organization, Nervous System Development and Function and (ii) RNA Post-Transcriptional Modification, Cell Cycle, Cellular Movement. The proteins attributed to the networks are shown in Table 2. The top 20 enriched biological functions are shown in Fig. 2D. The most represented category was the cellular assembly and organization group with growth of axons (*p* = 7.8 × 10^−7^), outgrowth of axons (*p* = 8.7×10^−7^), disassembly of filaments (*p* = 9.1 ×10^−6^), organization of cytoplasm (*p* = 1.1×10^−5^) and outgrowth of neurites (*p* = 1.87×10^−7^) as the top 5 most significant functions annotated. Another represented category was the RNA post-transcriptional modification group that is involved in splicing and processing of mRNA.

**Table 2:** Main Networks and the associated phosphoproteins that were regulated by CSPGs as analyzed by IPA from Ingenuity System.

Top Network Functions	Molecules	Score
Cell Morphology, Cellular Assembly andOrganization, Nervous SystemDevelopment and Function	ARMCX2, CRMP1, DBNL, DCX, DGCR14, DMD, DPYSL2, FLNC, KLC2, MAP1B,MAP2k4, MAP4, MSI2, MYO16, PAK4, PSMA6, REM2, RGL2, SRRT, SYN1, SYN3,SYNJ1, TAB2, ULK2, VIM	55
RNA Post-Transcriptional Modification,Cell Cycle, Cellular Movement	ARID1A, ATRX, BZW1, CARHSP1, CTNND1, DAB2IP, DOCK2, HIRIP3, MATR3,MLLT4, NCOA5, NDRG2, PABPN1, PRPF4B, SBF1, SMN1, SNW1, SNX17,SRC, SRRM1, SRRM2, SRSF2, THRAP3, TRA2B, TRIO	55

### Consensus Motif Mapping and Conservation Assessment of Phosphosites

**Figure 3:**
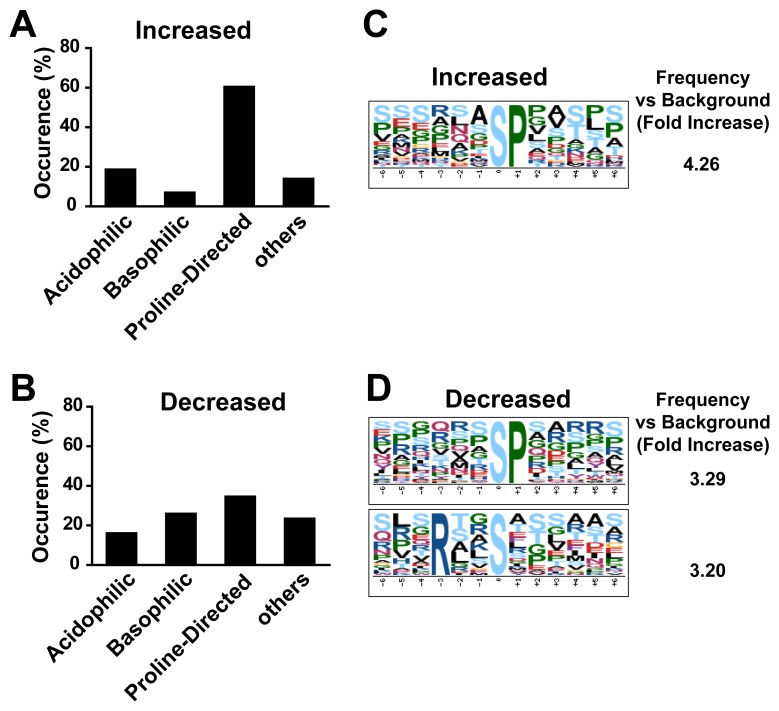
Motif analysis of significantly changed phosphopeptides. (**A**) Phosphorylation motif distribution for increased phosphopeptides by CSPG treatment. (**B**) Phosphorylation motif distribution for decreased phosphopeptides by CSPG treatment. (**C**) Sequence logos showing overrepresented phosphorylation motifs in 41 phosphopeptides significantly increase by CSPGs. (**D**) Sequence logos showing overrepresented phosphorylation motifs in 77 phosphopeptides significantly decreased by CSPGs.

Proline-directed, basophilic and acidiphilic are the three main Ser/Thr protein kinase groups classified based on the substrate residues that govern kinase-substrate recognition. The 43 phosphorylation sites from the 41 peptides with significantly increased phosphorylation and the 81 phosphorylation sites from the 77 peptides with significantly decreased phosphorylation were organized into these general sequence categories. Proline-directed phosphorylation (60.5%) was found dominant in the group with increased phosphorylation (Fig. 3A), while in the group with decreased phosphorylation, the frequencies of proline-directed, basophilic and acidiphilic sites were 34%, 25% and 16%, respectively (Fig. 3B). To better understand the consensus phosphorylation motifs induced by CSPG treatment, we performed phosphorylation motif analysis using software motif-X [21]. A proline-directed motif (P at +1) was found to be enriched in the increased group (Fig. 3C). One proline-directed motif (P at +1) and one basophilic motif with arginine residue at −3 were overrepresented in the decreased group (Fig. 3D).

High-throughput mass spectrometry has identified an overwhelming number of novel protein phosphorylation sites. However, the biological functions of most of these sites remain elusive. One alternative way to potentially address the functional relevance of a discovered phosphosite is to assess the evolutionary conservation of these sites across species since functionally relevant sites would be expected to be highly conserved [22]. We hence used CPhos [23], a program developed in our institute (available at: http://helixweb.nih.gov/CPhos/) to identify evolutionarily conserved functional phosphorylation sites [23]. We then evaluated the conserved sites using the homologene function at NCBI. The result of conservation analysis of the changed phosphosites is shown in Table S3, and demonstrated that 44% of the phosphosites were totally conserved amongst all sequences in homologene, 72% had greater than 75% conservation, and only 2% were conserved in less than 25% of the proteins. Thus, there is a high degree of conservation of the sites that are modified by CSPGs.

### Pathway Analysis

**Figure 4:**
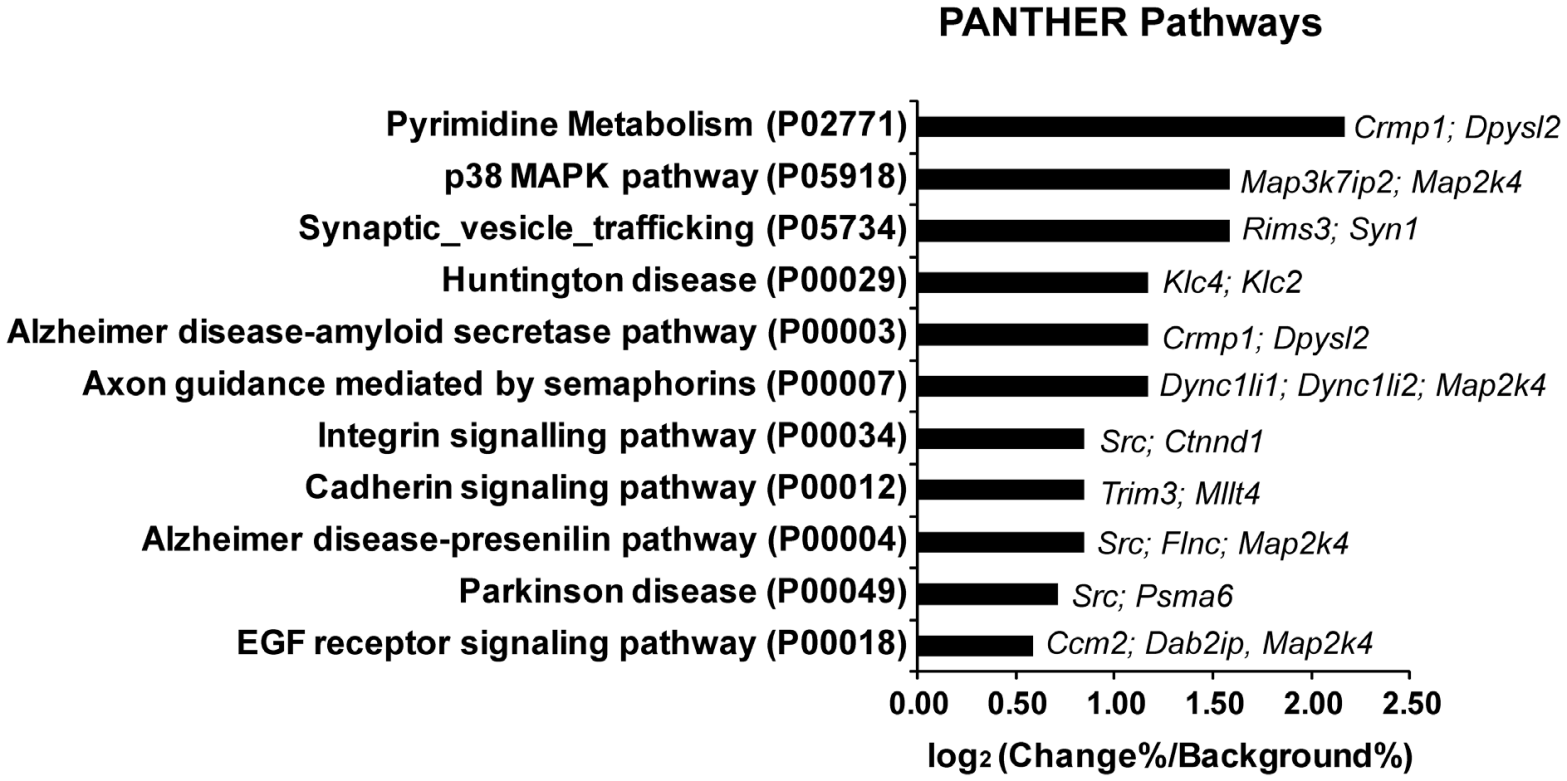
Signaling pathways regulated by CSPGs. PANTHER Pathway terms were extracted for each phosphopeptide that changed significantly with CSPGs and compared with pathway terms for all identified peptides which were used as background. The gene names for the corresponding phosphoproteins that changed with CSPGs are listed for each pathway. Only the pathways that contained more than one identified protein are included.

Analysis of PANTHER pathway terms for the 118 peptides whose phosphorylation was significantly changed revealed that a number of signaling pathways are regulated by CSPGs (Fig. 4). These included signaling pathways regulating pyrimidine metabolism, p38MAPK pathway, synaptic vesicle trafficking, Alzheimer’s disease-amyloid secretase, axon guidance mediated by semaphorins, cadherin signaling pathways, and the integrin signaling pathway which has previously been implicated in CSPG signaling [24]. Some of the phosphoproteins that are changed by CSPGs, such as Src and Map2k4, are shared among multiple signaling pathways, which suggests the existence of complicated crosstalk between signaling pathways.

### CSPGs Regulate Cofilin Phosphorylation

**Figure 5:**
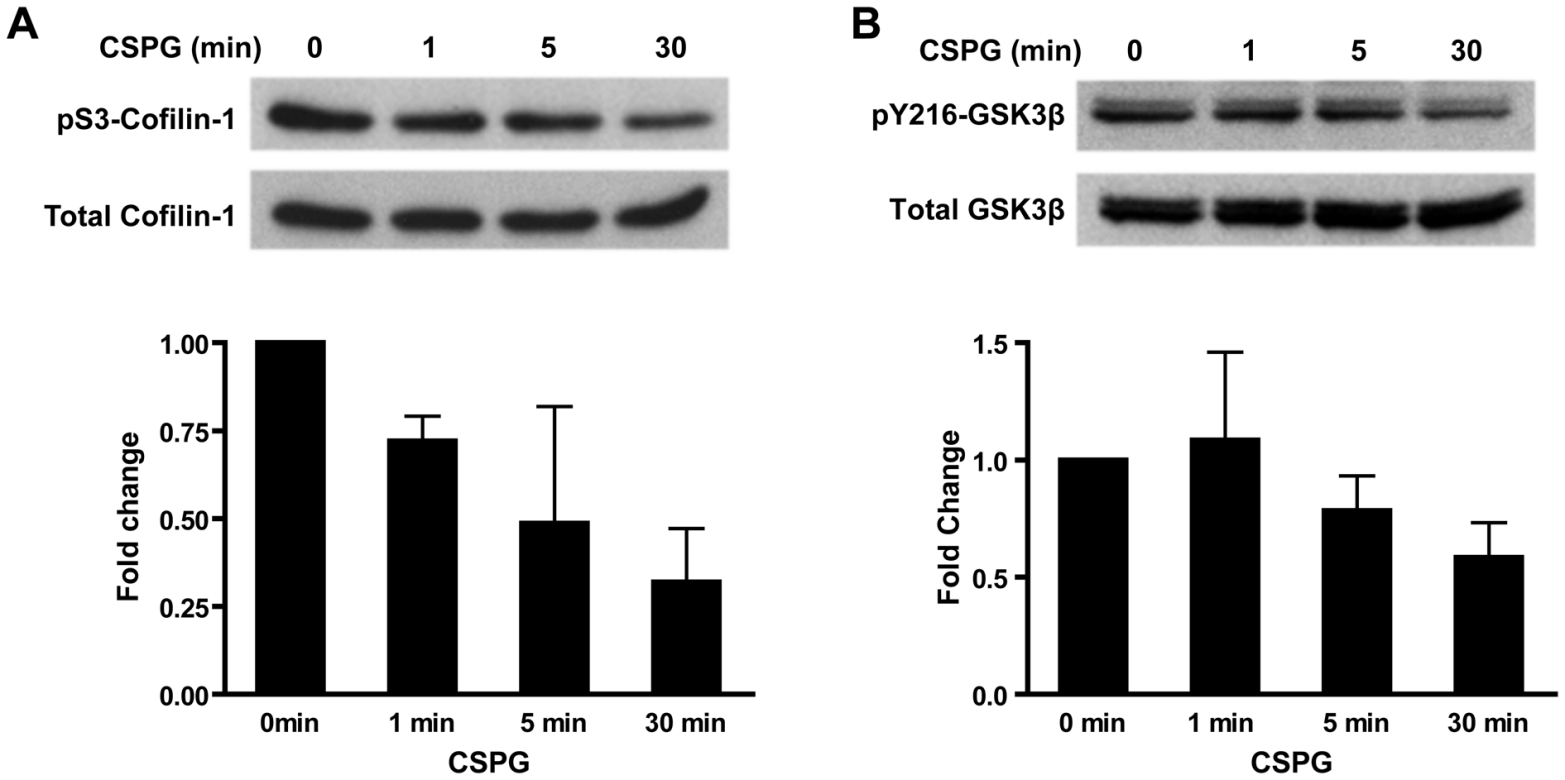
Western blot analysis of cofilin (pS3) and GSK3β (pY216) phosphorylation regulated by CSPGs. (**A**) The phosphorylation level of cofilin (pS3) and the amount of total protein expression at different time course after CSPG treatment; (**B**) The phosphorylation level of GSK3β (pY216) and the amount of its total protein expression at different time course after CSPG treatment.

Cofilin as an actin depolymerization factor has been implicated in growth cone motility and neurite outgrowth. The activity of cofilin is regulated by phosphorylation at serine-3. It has been shown that myelin-associated inhibitors, another class of neurite growth inhibitors, regulate cofilin phosphorylation in PC12 cells as well as primary CGNs [25]. In this phosphoproteomics experiment, two among the three biological replicates showed a decrease in cofilin phosphorylation at serine-3, with the third showed no change. The average phosphorylation level of these three replicates is decreased about 45% with a mean log_2_(CSPG/Control) value of −0.83 at 30 min after CSPG treatment. We performed another iTRAQ-8 plex phosphoproteomics experiment using the same procedure except without introducing SCX fractionation. In this experiment, the phospho-Serine3 of cofilin showed a consistent decrease among all three biological replicates, with a log_2_(CSPG/Control) value of −0.47, −0.87 and −0.33, respectively. To further confirm the regulation of cofilin phosphorylation by CSPGs, we examined the time course of cofilin phosphorylation in CGNs by western blotting with an antibody against Serine-3 phosphorylated (pS3) cofilin as well as an antibody against total cofilin. Cofilin phosphorylation was slightly decreased at 1 min and 5 min after CSPG treatment, and was further decreased at 30 min (Fig. 5A), the same time point as phosphoproteomics. This confirms that CSPGs promote cofilin dephosphorylation in primary neurons.

### CSPGs Regulate GSK3β Phosphorylation

GSK3β has also been shown play important roles in neurite outgrowth and axonal regeneration. Dill *et al*. showed that CSPGs regulated phosphorylation of an N-terminal serine residue Ser-9 of GSK-3β [18]; the levels of phosphorylated GSK-3β at Ser-9 negatively correlate with GSK-3β activity. In this study, we identified another two phosphorylation sites Ser-215 (pS215) and Tyr-216 (pY216), both of which showed a trend of decrease with a mean log_2_(CSPG/Control) value of −0.63 for pS215 and −0.30 for pY216, although the differences were not statistically significant likely related to the small sample size. To confirm these changes, we performed western blotting using an antibody against pY216 of GSK-3β and an antibody against total GSK-3β. The level of GSK-3β phosphorylation of pY216 was unchanged at 1 and 5 min after CSPG treatment, and modestly decreased by 30 min (Fig. 5B).

## Discussion

We have applied an iTRAQ-based quantitative phosphoproteomic strategy to investigate the signaling initiated by exposure to CSPGs in primary neurons. Overall, more than 2000 unique phosphopeptides were identified in CGNs, among which 118 phosphopeptides are changed in abundance upon CSPG treatment for 30 min. Phosphorylation is a complex and highly dynamic event involved in numerous biological processes. Although identifying changes in phosphorylation only at single time point is not enough to completely understand the temporal ordering or the whole picture of phosphorylation cascades, our studies use large-scale analysis to begin elucidate the players in signaling pathways. Our purpose is to screen protein candidates or pathways potentially affected by CSPGs, which then serves as the basis of further studies of CSPG signaling. By profiling the global phosphorylation changes using phosphoproteomics, this study has greatly expanded our knowledge about CSPG signaling.

The most overrepresented category among these changed phosphoproteins using the PANTHER protein classification was cytoskeleton binding proteins. A consistent result was obtained using the Ingenuity program, which identified Cell Morphology, Cellular Assembly and Organization, Nervous System Development and Function as the top one biological network. Among the top twenty significantly enriched biological functional categories, more than half were cytoskeleton related. Notably, growth of axons, outgrowth of axons and outgrowth of neurites showed the first, second and fifth highest significant enrichment, respectively. This is consistent with a conclusion that CSPGs exert many effects on axonal outgrowth and guidance through regulating the phosphorylation of cytoskeletal proteins involved in neurite outgrowth. We have already shown alterations in tubulin polymerization occur in response to CSPGs [26], and tubulin polymerization is known to be regulated by phosphorylation [27], [28].

Another category with significant enrichment was RNA-binding proteins involved in RNA posttranscriptional modifications. Changes in phosphorylation status of RNA binding proteins might significantly affect their activities and hence change the overall gene expression profile. Actually, a recent proteomic study showed that chondroitin sulfate compounds used in the treatment of osteoarthritis, altered the intracellular and extracellular proteome of human chondrocytes [29]. Most current studies of CSPGs on neurons have focused on growth inhibition, a relatively quick response which can be observed soon after neurons encounter CSPGs. It would be interesting to evaluate if these changes are dependent upon changes in gene or protein expression in addition to post-translational modifications.

CSPGs inhibit neurite outgrowth via two potential mechanisms: one might be a direct mechanism through CSPG receptors, another might be through indirect mechanisms by blocking the function of growth promoting ECM molecules and cell adhesion molecules, or by facilitating and presenting growth inhibitory proteins [30], [31], [32], [33], [34]. In our data, many pathways are shown to be affected by CSPGs; two of which are the cadherin and integrin pathways. It has been reported that interaction of neurocan, a major CSPG in the brain, with its GalNAcPTase receptor coordinately inhibits both N-cadherin and β1 integrin mediated adhesion and neurite outgrowth through increasing tyrosine phosphorylation of β-catenin [35], [36]. CSPGs have also been recently reported to inhibit laminin-mediated axon growth by impairing integrin signaling via decreasing tyrosine-861 phosphorylated FAK and tyrosine-418 phosphorylated Src levels [24]. All the above phosphorylation changes reported were on tyrosine residues, which are much less abundant than phosphoserine or phosphothreonine residues, accounting for <1% of phosphoamino acid residues in cells. In general, without specific enrichment of tyrosine phosphorylated proteins, global phosphoproteomic enrichment approaches using the IMAC method, as we applied in this study, to enrich phosphopeptides reveal small numbers of peptides containing phosphotyrosine [37]. Although we didn’t identify those phosphotyrosine sites in cadherin pathways or integrin pathways which have been shown regulated by CSPGs, we found other phosphorylation sites in these two pathways such as S74 of Src and T201 of catenin δ-1 that were significantly changed by CSPGs. CSPGs have been reported to inhibit neurite outgrowth of CGNs through inactivating Akt-Gsk3β signaling by transiently reducing the phosphorylation levels of pS473-Akt and pS9-GSK3β at 2 min after CSPG treatment followed by a rapid return to the control level by 5 min [18]. Due to the limitations of mass spectrometry-based peptide identification, the peptides containing those two phosphorylation sites were not identified in our experiments. However, we found another two phosphorylation sites of GSK3β (pS215 and pY216) were also regulated by CSPGs, although the function of this phosphorylation site is still unknown.

Another interesting pathway regulated by CSPGs was axon guidance mediated by semaphorins. The semaphorins are a family of secreted and membrane-bound glycoproteins that play important roles in axon guidance. Both Secreted Sema 3A [38] and membrane-anchored Sema 5A [30] have been shown to physically interact with CS-GAG chains. Our data further support the idea that binding of the GAG chain portion of CSPGs to guidance cues provides one of potential molecular mechanisms for CSPG-mediated axon growth inhibition. We have also identified new pathways regulated by CSPGs, such as p38MAPK pathway and synaptic vesicle trafficking pathway and some others, which provide new insights into the mechanisms by which CSPGs affect neuronal function.

iTRAQ which allows multiplexing of up to eight separately labeled samples within one experiment is currently one of the most widely applied techniques for large-scale quantitative proteomics analyses. However, one limitation that have been recently recognized and reported is ratio compression, leading to underestimation of the ratios [39], [40], [41]. Indeed, the changes observed in our iTRAQ experiment are modest and the overall ratios are compressed towards one with very few changed more than 2-fold. Therefore, we chose two phosphosites – pS3-cofilin and pY216-GSK3β – for western blot validation, which showed a trend towards decreased phosphorylation but did not reach *p*<0.05 in the iTRAQ analysis. We confirmed that phosphorylation of cofilin on serine-3 and phosphorylation of GSK3β on tyrosin-216 were both decreased 30 min after CSPG treatment. This implies that the phosphorylation changes in our iTRAQ results might also be underestimated as a result of ratio compression. Due to the lack of commercially available site specific-antibodies, we are not able to verify those significantly changed phophosites using western blot. Nevertheless, our results unmask several putative candidates as well as networks mediating CSPGs signaling on primary neurons, which can serve as a basis for the development of new strategies to overcome CSPG inhibition on axonal regeneration after CNS injury.

## Materials and Methods

### Ethics Statement

All animal experiments were carried out in compliance with the Guide for the care and use of laboratory animal resources (National Research Council, 1996) and approved by the National Heart, Lung, and Blood Institute Animal Care and Use Committee (NHLBI-ACUC, Protocol# H-0076).

### Cell Lysis

This study was carried out in strict accordance with the recommendations in the Guide for the Care and Use of Laboratory Animals of the National Institute of Health. Primary CGNs isolated from P5-8 day old mice were plated onto 10 cm poly-L-lysine (PLL)-coated dishes and cultured in neurobasal-A medium supplemented with B27 (1∶50, v/v). CSPGs (15 µg/mL, Millipore, Temecula, CA) or the same volume of PBS was added to the culture at day 2 and incubated for 30 min. Cells were then washed twice with PBS and lysed in 0.5 mL lysis buffer (8 M urea/50 mM Tris-HCl/75 mM NaCl) containing 1× protease inhibitor cocktail (Calbiochem) and 1× phosphatase inhibitor cocktail (Thermo Scientific) [42]. The cell lysates were sonicated immediately on ice with 0.5-s pulses for 30 cycles and spin at >10000 g for 10 min at 4°C. Supernatants were transferred into a new tube and protein concentration was determined by the bicinchoninic acid (BCA) assay. Approximately 400 µg of protein/sample was used for quantitative phosphoproteomic profiling. In order to ensure adequate coverage of phosphosites [43], 3 biological replicates (a total of 6 samples) including 3 control- and 3 CSPG-treated samples were collected from 3 independent cultures prepared from neonatal animals from three different litters.

### Proteolytic Digestion and Peptide Labeling by iTRAQ Reagents

Each protein sample was reduced with 10 mM DTT for 1 h, followed by alkylation with 40 mM iodoacetamide for 1 h under dark conditions. Samples were diluted with 50 mM ammonium bicarbonate to a less than 1 M final urea concentration before protein digestion with trypsin (Promega) at a mass ratio of 1∶20 (trypsin:protein) overnight at 37°C. Following tryptic digestion, peptide samples were desalted on Oasis HLB 1 cc (10 mg) Extraction cartridges (Waters, Milford, MA). The eluted peptides were dried in a SpeedVac and then labeled with iTRAQ reagents according to the manufacturer’s instructions. Briefly, the dried peptides were reconstituted in 80 µl dissolution buffer (20 µl/100 µg protein). Each of the six samples (Control-1, CSPG-1, Control-2, CSPG-2, Control-3, CSPG-3) was labeled separately with 4 vials of iTRAQ isobaric reagent (114, 115, 116, 117, 119 and 121), respectively. After incubation for 2 h at room temperature, the reaction was stopped by acidification with formic acid (1%). The 6 iTRAQ-labeled peptide samples were then combined and desalted on Oasis HLB1 cc (10 mg) Extraction cartridges (Waters). The eluted peptides were dried in a SpeedVac.

### Strong Cation Exchange Chromatography (SCX) of iTRAQ-Labeled Peptides

SCX fractionation was performed as previously described [42]. Briefly, dried iTRAQ-labeled peptides were resuspended in 300 µL of SCX buffer A (5 mM KH_2_PO_4_, 25% ACN, pH 2.67) and injected onto a PolySulfoethyl A SCX column (4.6 mm i.d. × 20 cm length, 5 *µ*m particle size, 300 Å pore size; PolyLC). SCX chromatography was carried out on an Agilent HP1100 system at 1 mL/min flow rate using the following gradient: 100% buffer A and 0% buffer B for 2 min; 0–14% buffer B for 33 min; 14–100% buffer B for 1 min; 100% buffer B held for 4 min (buffer B: 5 mM KH_2_PO_4_, 25% ACN, 500 mM KCl, pH 2.67). UV absorbance at 214 nm was monitored while fractions were collected every 1.5 min and pooled to create 26 fractionated samples. Each fraction was desalted on Oasis HLB 1 cc (10 mg) Extraction cartridges prior to phosphopeptide enrichment.

### Enrichment of Phosphopeptides by Immobilized Metal Affinity Chromatography

Phosphopeptide enrichment was performed using immobilized metal affinity chromatography (IMAC) with a Fe-NTA Phosphopeptide Enrichment (Thermo Scientific) according to the manufacturer’s instructions. Briefly, dried peptide samples were resuspended in 200 µL of binding buffer prior to loading onto Fe-NTA columns and incubated for 20 min with rotation. Columns were washed twice with 100 µL of wash buffer A, twice with 100 µL of wash buffer B followed by another wash with 100 µL of ultrapure water to equilibrate resin for elution. Elution of phosphopeptides was carried out using 50 µL of elution buffer each for three times.

Combined eluents were acidified with 100 µL of 2.5% trifluoroacetic acid. The samples were then desalted with the Pierce Graphite Spin Columns (Thermo Scientific) and resuspended in 0.1% formic acid prior to analysis by LC-MS/MS.

### LC-MS/MS Analysis and Database Search

Samples were analyzed on an Eksigent nanoflow LC system (Dublin, CA) connected to an LTQ-Orbitrap Velos mass spectrometer (Thermo-Fisher Scientific, San Jose, CA) [42]. iTRAQ labeled ions were fragmented by HCD (higher energy collisional dissociation) with normalized collision energy set at 45%. HCD fragmentation ions were detected in the Orbitrap at a resolution setting of 7500. MS2 spectra were searched with Proteome Discoverer software (ver.1.3, Thermo Scientific) running both the SEQUEST and the Mascot algorithms using the following criteria: database, Swiss-Prot, mouse; enzyme, trypsin; miscleavages, 2; static modifications, carbamidomethylation of cysteine (+57.021 Da), iTRAQ8-plex modification of peptide N-terminal (+304.205 Da), iTRAQ8-plex modification of lysine (+304.205 Da); variable modifications, oxidation of methionine (+15.995 Da); phosphorylation of serine, threonine and tyrosine (+79.966 Da); iTRAQ8-plex modification of tyrosine (+304.205 Da); precursor ion tolerance, 25 ppm; fragment ion tolerance, 0.05 Da. The dataset was filtered to give a <1% false discovery rate at peptide level using the target decoy method. The phosphorylation site assignment was performed using a dynamic programming algorithm PhosSA (http://helixweb.nih.gov/ESBL/PhosSA/) which has been optimized for HCD fragmentation [44].

### iTRAQ Quantification

Abundance ratios (CSPG/Control) were quantified by *Proteome Discoverer* and *Quari*, in-house algorithms for quantification of iTRAQ labeled peptides. To minimize contaminating near isobaric ions, only the peptides with isolation specificity more than 75% were quantified. For redundant peptides, the CSPG/Control ratio was calculated from the pair with the highest summed reporter ion intensity. The phosphopeptide ratios were normalized by dividing by the median ratio of all peptides identified. The log_2_ value of the ratio was used as the basis for the calculation of the mean and standard deviation (SD) for each peptide across all three biological replicates. An unpaired *t*-test was used to determine whether changes in phosphopeptide abundances were significant.

### Bioinformatics Analysis

Phosphoproteins that were identified were classified by the PANTHER classification system (http://www.pantherdb.org/). For the pathway analysis, the total identified phosphoproteins were used as background. The fold enrichment for each pathway term relative to the background was calculated by dividing the proportion of each Panther pathway term in the total changed phosphoproteins by the proportion of that particular Panther pathway term in the background data.

Ingenuity pathway analysis (IPA, http://www.ingenuity.com/) was used to identify protein networks and biological functions which are significantly over-represented in the list of changed phosphoproteins.

For phosphorylation motif analysis, the list of increased or decreased phosphopeptides was first aligned to 13-mer sequences with the phosphorylation site located in the center using an in-house program. Classification of phosphorylation motif was performed using a binary decision tree as previously described [45]: P at +1 (Pro-directed), 5 or more E/D at +1 to +6 (acidophilic), R/K at −3 (basophilic), D/E at +1/+2 or +3 (acidophilic), 2 or more R/K at −6 to −1 (basophilic), otherwise (others). The aligned phosphopeptide sequences were also submitted to the Motif-X algorithm (http://motif-x.med.harvard.edu/) to generate logos showing over-represented amino acids at specific positions relative to the phosphorylated amino acid.

### Western Blot

Dissociated CGNs were first plated on PLL-coated 6-well plates at a density of 1.5×10^6^ cells/well. At day 2, cells were treated with PBS or 15 µg/mL of CSPGs for 1 min, 5 min and 30 min. Cells were then washed twice with PBS and lysed with 2× SDS sample buffer in the presence of 1× phosphatase inhibitor cocktail (Thermo Scientific, Rockford, IL). The protein concentration was determined using the BCA assay. The cell lysates were then subjected to SDS-PAGE and immunoblotted with antibodies against phospho-cofilin (pS3) (1∶1000, Cell Signaling), cofilin (1∶1000, Cell Signaling), phospho- GSK3β (pY216) (1∶1000, BD Bioscience), and GSK3β (1∶1000, Cell Signaling). Horseradish peroxidase-conjugated secondary antibodies against rabbit (to detect cofilin) and mouse (to detect GSK3β) were used and visualized with an enhanced chemiluminescence (ECL) reagent.

## Supporting Information

Table S1 **List of all identified and quantified phosphopeptides.**
(XLSX)

Table S2 **List of phosphopeptides changed by CSPG treatment and its gene ontology analysis using the PANTHER classification system or Ingenuity program.**
(XLSX)

Table S3 **Conservation analysis of the changed phosphosites using CPhos program.**
(XLSX)
